# Amniotic fluid embolism as a cause of maternal mortality in China between 1996 and 2013: a population-based retrospective study

**DOI:** 10.1186/s12884-016-1106-6

**Published:** 2016-10-19

**Authors:** Yi Mu, Nolan McDonnell, Zhuoyang Li, Juan Liang, Yanping Wang, Jun Zhu, Elizabeth Sullivan

**Affiliations:** 1 National Office for Maternal and Child Health Surveillance of China, Department of Obstetrics, West China Second University Hospital, Key Laboratory of Birth Defects and Related Diseases of Women and Children, Ministry of Education, Sichuan University, Ren Min South Road Section 3 No.17, Chengdu, Sichuan China; 2 School of Women’s and Infants’ Health and School of Medicine and Pharmacology, University of Western Australia, Perth, Australia; 3 Faculty of Health, University of Technology Sydney, 235 Jones St, Ultimo, Sydney, NSW 2007 Australia; 4 Department of Anaesthesia and Pain Medicine, King Edward Memorial Hospital for Women, 374 Bagot Road, Subiaco, WA 6008 Australia

**Keywords:** Amniotic fluid embolism, Maternal mortality ratio, Maternal mortality, Maternal death, Pregnancy, China

## Abstract

**Background:**

To analyse the maternal mortality ratio, demographic and pregnancy related details in women who suffered a fatal amniotic fluid embolism (AFE) in China.

**Methods:**

A retrospective population based study using data collected as part of the National Maternal Mortality Surveillance System between 1996 and 2013. Data were collected onto a standardised form from women whose cause of death was listed as being secondary to AFE.

**Results:**

Records were available for 640 deaths. Over the 17 year period the maternal mortality ratio for AFE decreased from 4.4 per 100,000 births (95 % confidence interval (CI):2.72–6.12) to 1.9 per 100,000 births (95 % CI:1.35–2.54). Over the same period the proportion of maternal deaths secondary to AFE increased from 6.8 to 12.5 %. The mean age of women who died was 30.1 years and the onset of the AFE occurred prior to delivery in 39 %. The most prominent presenting features included premonitory symptoms (29 %), acute fetal compromise (28 %), maternal haemorrhage (16 %) and shortness of breath (15 %).

**Conclusions:**

Maternal mortality secondary to AFE has decreased in China, however at a slower rate than mortality secondary to other conditions. Active surveillance is recommended to assess case fatality rates, risk factors and other lessons specific to this population.

## Background

In September 2000 the United Nations Millennium Declaration committed world leaders to improving the health and wellbeing of member states by the year 2015 through a number of specific Development Goals [1]. Millennium Development Goal 5 (MDG5) aims to improve maternal health and more specifically, Target 5.A. aims to reduce the maternal mortality ratio (MMR) by three quarters for the time period 1990–2015 [2]. Currently, approximately 290,000 women die each year from pregnancy related complications [3]. Throughout the world there are significant differences in the MMR secondary to a number of factors including the underlying socioeconomic state of the population, geographical location, access to contraception and access to skilled birth attendants [3–5].

China has had well documented success in reducing their maternal mortality in line with MDG5 [6]. This has been in large part secondary to the transition to women giving birth in a hospital environment [7]. This has been supported by widespread media campaigns, subsidies to promote hospital based birth, infrastructure improvements as well as increased training for staff involved in the care of pregnant women [8, 9]. The majority of the decrease in maternal mortality can be explained by a reduction in more potentially preventable causes of mortality such as haemorrhage, hypertensive disorders of pregnancy and sepsis [10].

Amniotic fluid embolism (AFE) is a rare complication of pregnancy with a comparatively high mortality [11]. It often presents as the sudden onset of cardiovascular collapse, respiratory compromise and disseminated intravascular coagulation. AFE is a poorly understood condition whose management requires prompt and timely access to providers with training in maternal resuscitation as well as resources to manage the potential cardiovascular, respiratory and haematological compromise [11]. In contrast to developing nations, in well-resourced countries such as Australia, New Zealand and the United Kingdom AFE is a leading cause of maternal mortality [12–14]. However, there has been little attention focused on AFE as a contributor to maternal mortality in developing nations.

As countries continue to work towards reducing their maternal mortality, conditions such as AFE are likely to become more prominent as other more preventable causes decline in frequency. The aim of this present study was to analyse the MMR, demographic and pregnancy related details in women who suffered a fatal AFE in China between January 1 1996 and September 30 2013.

## Methods

This study was designed as a population based study using data collected as part of the National Maternal Mortality Surveillance System (NMMSS). The NMMSS is part of the National Maternal and Child Health Surveillance System (MCHSS) and was established by the Chinese Ministry of Health in 1989. The process of the establishment of the NMMSS and the methods of data collection have been described in detail elsewhere [10]. From 1996 to 2006, the MCHSS covered 176 surveillance districts/counties representing approximately 80 million people with an annual number of live births ranged from 493,662 to 622,856. After 2006, the MCHSS was expended to 336 surveillance districts/counties covering approximately 140 million people with an annual number of live births ranged from 1,759,539 to 2,111,365.

For the purposes of the NMMSS a maternal death is classified in line with the World Health Organization (WHO) definition [15] and is the death of a woman while pregnant or within 42 days of termination of pregnancy, irrespective of the duration and of the pregnancy, from any cause related to or aggravated by pregnancy or its management, but not from accidental or incidental causes.

A standardised procedure is used to determine the cause of death for each case that each maternal death is reviewed by four levels of committees (county/district review committee, municipal review committee, provincial review committee and the National Review Committee) [16]. If a maternal death occurs, the death is investigated by trained obstetricians including interviews of the family, midwives, physicians and nursing staffs. The information were collected using a standardised form on prenatal care; history of the pregnancy; course of delivery; the date of delivery and death; cause of maternal death and details of the treatment provided. This information is then reviewed by a county/district review committee to determine the cause of death and identify factors contributing to death. A maternal death secondary to AFE is defined either as a clinical diagnosis (acute hypotension or cardiac arrest, acute hypoxia, acute pulmonary embolism, cardiovascular collapse, disseminated intravascular coagulation, renal failure and a series of pathological changes in the absence of any other potential explanation for the symptoms and signs observed) or as a post mortem diagnosis (presence of fetal debris in the circulation). The municipal and provincial maternal and child health institutions review and verify the investigative reports. Finally, committees at the provincial and national level re-examine the results submitted by the subordinate committee and make the final decision as to the likely cause of death. The ascertainment rate of maternal death was approximately 96 % for all causes of death.

For the purposes of this study the NMMSS database was examined for maternal deaths between January 1 1996 and September 30 2013 where AFE was recorded as the cause of death. Data was then extracted from the mortality records using a standardised data collection form based on those originally developed by the Australasian Maternity Outcomes Surveillance System (AMOSS) [17], and then modified by the National Office for Maternal and Child Health Surveillance. The modified survey form included baseline demographic and pregnancy factors, obstetric interventions, the location of the woman’s initial presentation/collapse, the most prominent initial presenting feature, other associated features, therapy required, results of relevant investigations and maternal and perinatal outcomes. Premonitory symptoms are defined as those present before the abrupt change in maternal condition that led to the diagnosis of AFE and may include: breathlessness, chest pain, feeling cold, light headedness, restlessness, distress, panic, a feeling of pins and needles in the fingers, nausea and vomiting. A stillbirth was defined in accordance with WHO criteria, that being a baby born with no signs of life after 28 weeks gestation. A neonatal death was defined as death occurring within the first 28 days post-delivery.

### Statistical methods

The MMR was calculated from the number of maternal deaths per 100 000 live births. The 95 % confidence intervals (CI) of the MMR in urban and rural areas were calculated by assuming that the counts follow a Poisson distribution. We did statistical analyses with SPSS software (version 16.0; SPSS Inc., Chicago, IL, USA).

## Results

**Fig. 1:**
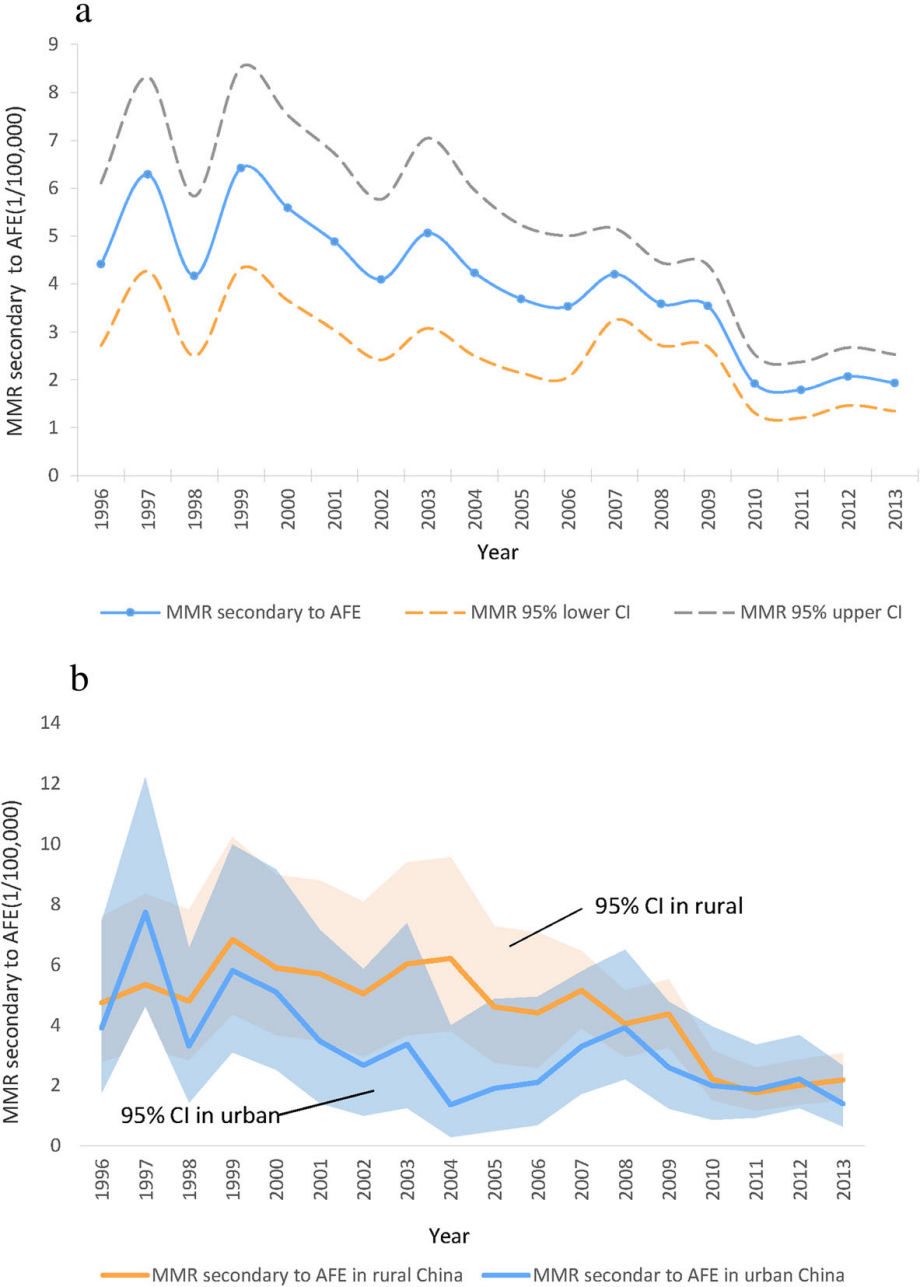
**a** Maternal mortality secondary to amniotic fluid embolism in China, 1996–2013. **b** Maternal mortality secondary to amniotic fluid embolism in urban and rural China, 1996–2013

**Fig. 2:**
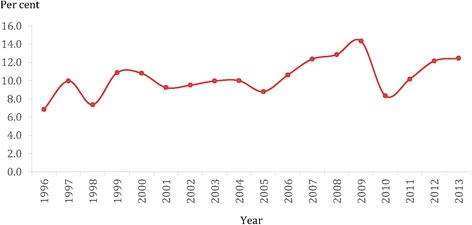
The proportion of of maternal deaths secondary to amniotic fluid embolism in China, 1996–2013

Over the course of the study period there were 664 recorded deaths secondary to AFE, of which summary medical records were available for 640 cases. Over the corresponding period there were 20,006,880 live births, giving an estimated maternal mortality from AFE of 1:30,131 births (95 % CI: 1:26001–1: 32611 births). The MMR for AFE over the study period decreased from 4.4 per 100,000 (95 % CI 2.72–6.12) in 1996 to 1.9 per 100,000 (95 % CI 1.35–2.54) in 2013 (Fig. 1a). Over the corresponding period the proportion of maternal deaths secondary to AFE increased from 6.8 % in 1996 to 12.5 % in 2013 (Fig. 2).

The demographic details of the women who died are shown in Table 1. The mean age of the women who died was 30.1 (±5.4) years. Eighty four percent of pregnancies were singleton. The onset of the AFE episode occurred prior to delivery in 39 % and after delivery in 53 % (unknown in 8 %) (Table 2). For those cases that occurred postpartum, 78 % occurred in the first 60 min after delivery (56 % in the first 30 mins, 22 % between 30–60 min after delivery). The location of the AFE episode was recorded as being at home in 8.4 % of cases, in the delivery suite in 36 % of cases, the operating theatre in 13 % and the maternity ward in 14 %. Twenty four percent of women did not give birth whilst 60 % had a vaginal birth and 16 % a caesarean delivery.

**Table 1:** Demographic and obstetric characteristics of the maternal deaths secondary to amniotic fluid embolism in China, 1996–2013

Demographic and obstetric characteristics	Number	Percent
Age (year) Mean (SD)	30.1 (5.4)	
Age (year)		
< 25	85	13.3
25–29	158	24.7
30–34	227	35.5
> =35	170	26.5
Ethnicity		
Han	557	87.0
Other	83	13.0
Education		
Literate	35	5.5
Primary	169	26.4
High	396	61.9
Collage or above	35	5.5
Unknown	5	0.8
Parity		
Primiparas	309	48.3
Multiparas	329	51.4
Unknown	2	0.3
Number of gestations		
Singleton	539	84.2
Twins	14	2.2
Three or more	12	1.9
Unknown	75	11.7
Number of antenatal visits		
0	53	8.3
1	36	5.6
2–4	218	34.1
> =5	305	47.7
Unknown	28	4.4

**Table 2:** Delivery and collapse details of the maternal deaths secondary to amniotic fluid embolism in China, 1996–2013

Delivery and collapse details	Number	Percent
Rupture of membranes prior to onset		
Yes	504	78.8
Artificial	100	15.6
Spontaneously	315	49.2
Unknown	89	13.9
No	136	21.3
Meconium stained liquor		
None	79	12.3
Fresh	60	9.4
Old	101	15.8
Unknown	400	62.5
Method of birth		
Undelivered^a^	154	24.1
Non-instrumental vaginal	290	45.3
Instrumental vaginal	93	14.5
Caesarean	103	16.1
Location of onset of symptoms		
Home	54	8.4
Maternity ward	91	14.2
Labour room	6	0.9
Theatre	85	13.3
Delivery Suite	227	35.5
Other	28	4.4
Unknown	149	23.3
Pregnancy state at onset of collapse		
Pre-labour	30	4.7
First stage	112	17.5
Second stage	111	17.3
Post delivery	337	52.7
Unknown	50	7.8
Time from delivery until onset of symptoms^b^		
0 ~ 0.5 h after delivery	141	55.73
0.5 ~ 1 h after delivery	55	21.74
1 ~ 1.5 h after delivery	23	9.09
1.5 ~ 2 h after delivery	8	3.16
2 ~ hours after delivery	26	10.28
Place of death		
Hospital	554	86.6
City	169	26.4
County	261	40.8
Township	121	18.9
Village	3	0.5
Home	39	6.1
On the way to hospital	42	6.6
Other	5	0.8

^a^Including termination of pregnancy

^b^If delivered prior to onset of symptoms

**Fig. 3:**
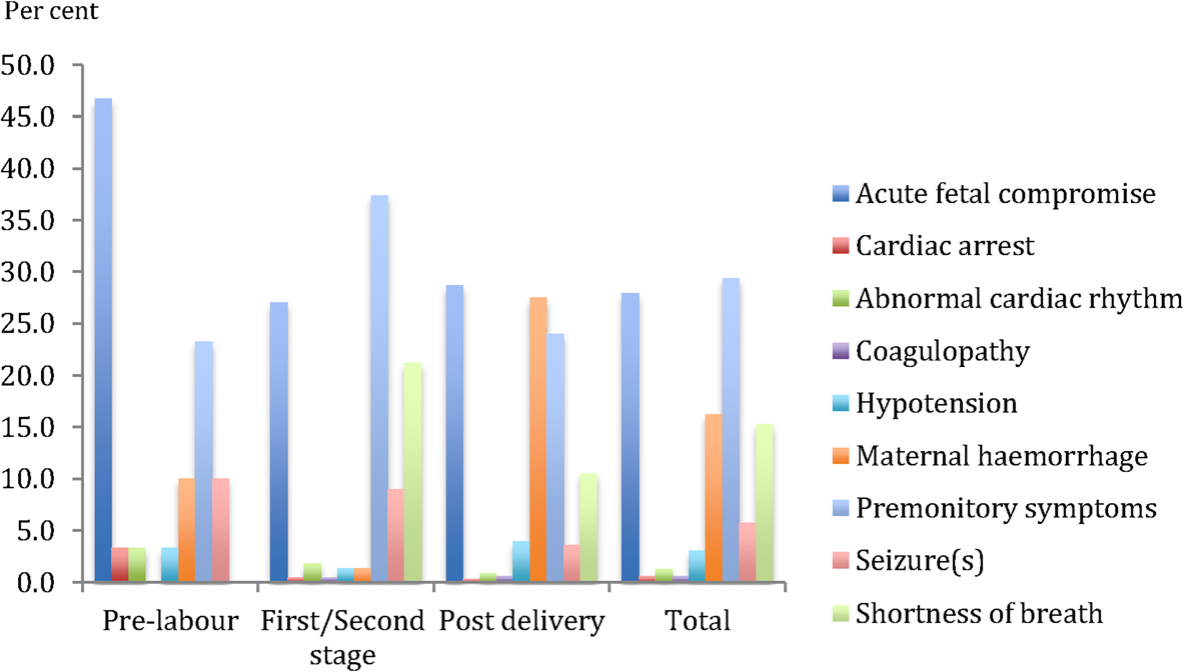
The single most prominent initial presenting feature by pregnancy state at onset of collapse of maternal deaths secondary to amniotic fluid embolism in China, 1996–2013

**Fig. 4:**
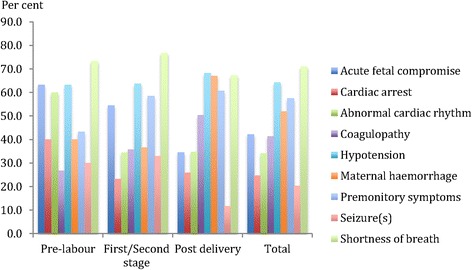
Overall presenting features by pregnancy state at onset of collapse of maternal deaths to amniotic fluid embolism in China, 1996–2013

The most prominent initial presenting feature and the associated presenting features varied by the pregnancy state at the onset of the collapse (Figs. 3 and 4). Overall, premonitory symptoms were the most commonly described initial presenting feature (29 %) followed by acute fetal compromise (28 %), maternal haemorrhage (16 %) and shortness of breath (15 %).

**Fig. 5:**
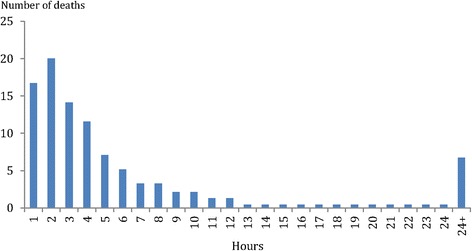
Time from the onset of the amniotic fluid embolism episode until death, maternal deaths secondary to amniotic fluid embolism in China, 1996–2013

Details of the treatment required are shown in Table 3. Cardiopulmonary resuscitation was performed in 75 % of women whilst 40 % required intubation and ventilation. Thirty seven percent of women were administered blood or blood products and a hysterectomy was performed in 18 %. The time from the onset of the AFE episode until death was confirmed is shown in Fig. 5. An autopsy was performed in 43 women (6.7 %) and in 10 (23.3 %) there was documented evidence of material consistent with amniotic fluid in the maternal pulmonary circulation.

**Table 3:** Treatment required during the management of the maternal deaths secondary to Amniotic fluid embolism in China, 1996–2013

Treatment	Number	Percent
Cardiopulmonary resuscitation	478	74.7
Intubation/Ventilation	258	40.3
Hysterectomy	113	17.7
Recombinant Factor VIIa	32	5.0
Blood and blood products	239	37.3

Only limited neonatal data were able to be extracted from the NMMSS. Twenty four percent of women did not give birth (died undelivered), for the remaining 76 % of women (*n* = 486) information was only available for 160 of which there were 91 stillbirths, 30 neonatal deaths and 39 neonates surviving past 27 days of age.

## Discussion

Between 1996 and 2013 there were 664 deaths classified as being secondary to AFE in the regions of China that participated in the NMMSS maternal mortality surveillance. Over this time period the MMR secondary to AFE declined from 4.4 to 1.9 per 100,000 births however the proportion of maternal deaths secondary to AFE increased from 6.8 % in 1996 to 12.5 % in 2013. Although the proportion of maternal deaths secondary to AFE has increased over this period, the absolute numbers have decreased most likely reflecting the more rapid decline in deaths secondary to more preventable and common causes such as haemorrhage.

The major strength of this study is the large number of maternal deaths secondary to AFE. The 664 deaths reported here represent internationally the largest cohort of maternal deaths to have been reported. As the data was collected over a 17 year time period and across a broad geographical area, it provides contemporary information on the trends in MMR from AFE over this time period and also the differences across urban versus rural China. However, as data for these cases was collected retrospectively from a standardised dataset, only limited data that was routinely entered as part of the NMMSS data collection process was available for review. Population based data collection without the use of additional criteria to exclude false positive cases may lead to an overestimation of the true incidence [18]. As there is currently no active surveillance of AFE in China it is not possible to report a case fatality rate, overall incidence of AFE as well as risk factors for AFE and factors associated with survival from AFE. Data in relation to fetal outcomes was limited by a lack of routine neonatal data collection in the NMMSS. As AFE is a diagnosis of exclusion [19], maternal autopsy is important to help exclude other potential causes of collapse and it has been a consistent message from the UK Confidential Enquiries that access to high quality pathology services is required for maternal deaths [14, 20] and guidelines for the performance of a maternal autopsy are available [21]. Of the 664 deaths reported here, only 43 (6.5 %) women underwent a post mortem examination, potentially secondary to a historical lack of access to pathology services. This low rate of post mortem examination may lead to higher numbers of both false positive and false negative diagnoses and suggest that efforts to further improve the maternal mortality in China should also be done in conjunction with efforts to increase the access to and uptake of maternal autopsy.

The decrease in the MMR from AFE in this population is likely to be secondary to the considerable efforts focused on improving maternal mortality in China since the establishment of the Millennium Development Goals. In line with MDG5, the Chinese government invested considerable resources in decreasing maternal mortality. Projects such as the “Reducing Maternal Mortality and Eliminating Newborn Tetanus” have aimed to promote hospital based births for rural women as well as improve resources and training of staff [22]. However, despite the decrease, the MMR reported here is similar to other developing countries (ranged between 1.8–5.9 per 100,000 deliveries) [23] and higher than in many developed countries. The most recent triennial confidential enquiry into maternal deaths in the UK and Ireland reports an MMR from AFE of 0.33 per 100,000 [14] whilst data from Australia reports an MMR of 0.6 per 100,000 [12]. A number of factors may explain these differences, including socio-economic factors as well as timely access to skilled health care providers in the event of maternal compromise. Previously, the MMR in rural versus urban areas in China has shown significant disparity [10] with a larger number of more preventable deaths [16]. This gap between rural and urban areas now appears to be narrowing [24] and in the data presented here, the MMR for AFE is now similar for both rural and urban areas.

In this study, the most prominent initial presenting feature differed depending on the pregnancy state of the mother. Previous studies have generally not differentiated the presenting features in relation to the pregnancy state and in this regard the data presented here provides useful information that may assist with more timely recognition of an AFE episode. Women whose onset of the AFE occurred in utero showed a high incidence of acute fetal compromise as the initial presenting feature, most likely because the utero-placental unit is often very sensitive to changes in the maternal haemodynamic state. The development of acute fetal compromise, especially in the presence of abnormal maternal signs and symptoms, should alert the care provider of the potential for impending maternal deterioration and potentially prompt the mobilisation of additional resources.

Currently in China there is no widespread system in place to monitor rare conditions of pregnancy. Obstetric surveillance systems such the AMOSS and the UK Obstetric Surveillance System (UKOSS) have demonstrated the benefit that these systems can have in furthering the understanding of rare conditions of pregnancy [25] as well as in rapidly responding to emerging issues that can impact on pregnant women [26]. In relation to AFE, the lack of an active surveillance system means potentially important clinical and health systems information specific to the Chinese population and maternity care context regarding risk factors for AFE, comparisons of women who survive and don’t survive their AFE as well as overall case fatality rates are not able to be reported. Given the comparatively high MMR for AFE reported here, the establishment of a surveillance system for AFE and other rare conditions of pregnancy is likely to be a worthwhile investment, not only in China, but also in other developing nations who have undergone significant reductions in maternal mortality.

## Conclusions

The MMR secondary to AFE in a large Chinese population declined by over 50 % in the 17 year period from 1996–2013. Over this same time period and as a likely consequence of a reduction in deaths from more preventable causes, the proportion of maternal deaths secondary to AFE increased. The likely reasons for the decline in the mortality ratio from AFE include the greater urbanisation of the Chinese population in conjunction with improved health care in more disadvantaged regions. As other developing nations are likely to go through similar changes in the risk profile of maternal deaths, there are a number of potential lessons to be learned. To more comprehensively study rare disorders of pregnancy, the use of surveillance systems similar to that used in Australia/New Zealand (AMOSS) and the UK (UKOSS) potentially provide more useful information then that derived from conventional mortality reporting. As AFE may present suddenly and require prompt and significant multi-disciplinary resources to effectively manage, the provision of appropriate training and infrastructure in delivery settings is important to improve outcomes and may require significant investment in more isolated regions.

## Abbreviations

AFE: Amniotic fluid embolism
MDG5: Millennium development goal 5
MMR: Maternal mortality ratio
NMMSS: National Maternal Mortality Surveillance System
MCHSS: National Maternal and Child Health Surveillance System
WHO: World Health Organization
AMOSS: Australasian Maternity Outcomes Surveillance System
CI: Confidence intervals
UK: the United Kingdom of Great Britain and Northern Ireland
UKOSS: UK Obstetric Surveillance System
